# Prehospital emergency medical service missions to nursing homes – a descriptive retrospective register-based study in Southwest Finland

**DOI:** 10.1186/s12873-026-01631-7

**Published:** 2026-05-30

**Authors:** Henna Myrskykari, Timo Iirola, Hilla Nordquist

**Affiliations:** 1 University of Helsinki Faculty of Medicine, Helsinki, Finland; 2 Emergency Medical Services, Wellbeing Services County of Southwest Finland, Turku, Finland; 3 University of Turku, Turku, Finland; 4 Department of Health and Social Management, University of Eastern Finland, Kuopio, Finland; 5 Department of Sustainable Wellbeing, South-Eastern Finland University of Applied Sciences, Mikkeli, Finland

**Keywords:** Emergency medical services, Nursing home, Paramedics

## Abstract

**Background:**

Residents of nursing homes often need emergency medical services or visits to the emergency department, due to deterioration of their general condition or to trauma. However, some of these situations could be treated in nursing homes. This study examined the reason for calling emergency medical services to nursing homes and the outcomes of these missions.

**Methods:**

This was a descriptive retrospective register study of emergency medical service missions of 510 older adults in 106 nursing homes in Southwest Finland, from September 1, 2023 to January 31, 2024. Emergency medical service reports and related emergency department visits were analyzed descriptively.

**Results:**

In total, 633 emergency medical service (EMS) missions were included in the study: 581 were on-scene missions and 52 missions were managed remotely. The most common cause reported for a mission was a fall (*n* = 169), followed by deterioration of the patient’s general condition (*n* = 89) and shortness of breath (*n* = 73). In 60.5% of EMS missions, some treatment procedures had been performed prior to the emergency call, 60.3% of patients visited the emergency department and 20.8% needed follow-up treatment.

**Conclusions:**

The majority of nursing home missions are non-urgent, and only some patients require follow-up treatment after an emergency department visit. Prospective studies are needed to determine whether outreach acute care services and mandatory advance care planning (ACP) can reduce unnecessary missions and transfers.

## Introduction

The condition of multimorbid older adults living in a nursing home can deteriorate, for example, due to infections, changes in medication, traumas and exacerbations of chronic diseases, as well as acute medical emergencies [1, 2]. Nursing home staffs’ ability and guidelines for handling different situations vary greatly [3], and not all units have their own physician or registered nurses constantly available [4–6]. In these situations, advice is often asked from the emergency department (ED) or by alerting emergency medical service (EMS) [7].

In recent years, there has been a significant increase in the number of EMS missions alerted to care facilities [8], and EMS are also alerted to care for less acute situations [9]. A previous study found that EMSs are alerted up to four times more often for older adults living in nursing homes than for older adults living at home [10], and older adults are also about twice as likely to require EMS physician care compared with individuals aged 18 to 65 [11]. However, previous research findings have not been entirely consistent. Surprisingly, Hancock et al. [12] found that emergency calls were more likely to be made from nursing homes with higher quality and suitability of management, as well as from nursing homes with specialization in dementia patients. According to Lofthouse-Jones et al. [13], the presence of dementia as a condition did not increase the likelihood of transfers, but patients living in care facilities were more likely to be transferred to a hospital, whereas in the study by Suzuki et al. [14], nursing home residence was not identified as an independent predictor of hospital admission.

In previous studies, the majority (78–90%) of the EMS missions of nursing homes ended up with patients being transferred to a hospital [8, 15–18]. However, it has been suggested that some of the transfers may be unnecessary [19, 20], and that unjustified transfers could be prevented if the patient’s Do Not Resuscitate (DNR)-decisions and treatment limitations were available [17]. Unnecessary transfers burden older adults and add avoidable complications [19, 21, 22], while also burdening the health care system. Therefore, the necessity and potential benefits of hospital transfers should be carefully evaluated in each case.

Comprehensive studies examining treatments provided by nursing home staff prior to the EMS arrival and the activities of the EMS personnel have not been previously investigated in Finland, and are also quite scarcely studied globally. However, this information is needed to develop practices. In this descriptive study, with no pre-specified hypotheses, the objectives were:

1. Examine the reasons for calling EMS to the nursing home.
2. Describe treatment procedures followed prior to the arrival of EMS.
3. Analyze treatment measures by EMS.
4. Analyze outcomes of the call.

## Methods

The study was a descriptive retrospective register-based study including all 24-hour staffed care facilities for the older adults in Southwest Finland. The data was collected from September 1, 2023 to January 31, 2024.

### Setting

In Finland, wellbeing services counties are responsible for the health and social services. However, housing services for older adults can be organized by wellbeing service counties, private service providers or associations. Housing services are divided into communal housing and 24-hour services like nursing homes and long-term residential care facilities. In the 24-hour housing service, nursing staff are constantly present, while in communal housing like sheltered housing, nursing care is typically provided through home care visits. In Finland, a person is entitled to 24-hour housing service in situations where the person needs 24-hour care and is not able to live in their own home with the help of home services.

In the 24-hour services, the staff usually consists of care assistants, practical nurses and registered nurses. According to the general perception, there are major differences in the ability and guidelines of the units to treat the deterioration of the residents’ condition. If a given facility has no physicians of its own available, the nurses can ask for advice from ED.

Wellbeing service counties are responsible for organizing EMS in Finland. In Finland, there is a single emergency number, 112. The dispatchers of the emergency response centers are not health care professionals, and they assess the need for EMS according to national guidelines. If necessary, they forward the mission directly to an ambulance or EMS telephone service. In non-urgent situations, dispatchers can also instruct the person to call an out-of-hours medical helpline.

The wellbeing services county of Southwest Finland has a population base of 494 819 [23], and its EMS includes ambulances, an EMS telephone service, a physician-staffed helicopter unit, and a helicopter unit of the Finnish Border Guard. The EMS telephone service can contact patients with regard to non-emergency missions, prior to ambulance dispatch, during daytime hours. The EMS is thus able to handle some missions remotely without dispatching an ambulance to the scene. In the study region, ambulances are staffed (as a rule) by one advanced-level paramedic and one basic-level paramedic. Advanced-level paramedics have either a four-year dual bachelor’s degree in emergency care and nursing, or a Bachelor of Nursing degree with advanced-level out-of-hospital specialization. Basic-level paramedics have 2–3 years of vocational education [24]. In Finland, paramedics are allowed to make relatively independent decisions on missions. According to national and regional treatment guidelines, patients do not always need to be transferred to an ED by ambulance since many cases can be treated at the scene or the patient can be transferred to the ED by taxi. On the scene, paramedics can, for example, administer inhaled bronchodilator therapy or pain medicine, treat a nosebleed, glue wounds, or arrange other help. Depending on regional guidelines, paramedics may also be able to make a decision not to transfer the patient without consulting a physician if the patient’s vital signs are normal or if the patient has not had a risk symptom.

### Data collection procedures

**Fig. 1:**
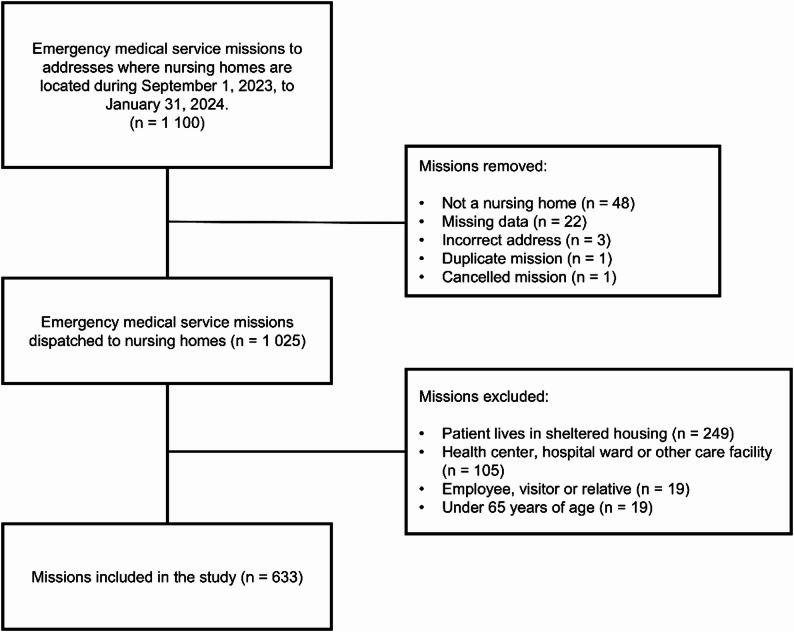
The data identification and selection process from electronic patient care reports

The electronic patient care reports (ePCRs) of the EMS and ED of the Wellbeing Services County of Southwest Finland were used. All EMS missions in the four different urgency classes were included. In Finland, Class A and B missions are urgent (i.e., “lights and siren”) missions. A Class A mission is a high-risk mission in which the patient has been assessed as having serious, immediate need for treatment in regard to basic vital functions. A Class B mission is probably a high-risk mission, but the disorder of basic vital functions has not been confirmed. For non-urgent missions, classes C and D, the patient should be reached within 0.5 h and 2 h, respectively. In Class C, the patient’s condition is stable or the disorder of basic vital functions is minor, but the patient needs an on-call assessment. In Class D the patient does not have a disorder of basic vital functions but needs an assessment of the need for treatment by EMSs. All EMS missions alerted to 24-hour housing service units, and where the resident was 65 years of age or older, were included. Also missions handled remotely by EMS were part of the sample. No prior sample size calculation was performed because the study aimed to include all eligible EMS missions to qualifying nursing homes during the study period. The sample excluded missions for persons who lived in sheltered housing and only used home care services, or who were residents of a 24-hour housing service unit due to an intellectual disability or a mental health or substance abuse problem. The type of housing was exploratively interpreted from documents by excluding missions where EMS or ED personnel mentioned *‘a home care visits X time per day/week’*, *‘home care client’*, *‘home care contacted EMS’* or *‘living alone*,* no home care services’*. In missions where documentation was ambiguous or did not contain relevant information, the mission was classified as excluded if the address also had sheltered housing, and the patient or a relative had made an emergency call, and the receiving hospital’s electronic health record (EHR) system made no mention of living in a nursing home. Based on these criteria, 249 missions were excluded. The data identification and selection process from ePCRs is presented in Fig. 1. The gathered data was pseudonymized, and the analyses were conducted using the SD Desktop service provided by CSC - IT Center for Science, Finland.

### Recorded data

EMS missions, their dispatch codes and timestamps, and events recorded on the mission were collected. Background information on patients, such as DNR orders and primary diseases, were collected from EHR system and, in addition, from the referrals of nursing homes saved in ePCRs of ED. Advance care plans that included treatment guidelines and treatment methods [25], as well as treatment limitations, were also collected from ePCRs of EMS, if these were described in more detail in the documents. If they were not mentioned, these variables were assumed to be absent. The patient was reported as having dementia or decline in memory function if this had been diagnosed, if a Mini-Mental State Examination score of 23 or below had been documented, or if the involved physician had documented a decline in memory function.

The reasons for calling EMS were interpreted from paramedic documentation and classified using the EMS mission codes of the Finnish Emergency Response Center. The urgency of the mission was reported directly according to the urgency class dispatching by the Emergency Response Centre, and the urgency of the transfers was assessed based on the documentation of paramedics. EMS missions that were associated with a decrease in general condition were classified into three groups: gradual deterioration of general condition, sudden deterioration and fainting. Missions related to a fall included all missions where the suspected injury, pain, or nurse’s concern was clearly documented as related to the fall.

Information on treatment procedures implemented by nursing home staff was collected from the EMS and ED ePCRs. The patient’s symptoms at the time of the EMS encounter, their first vital signs, and the treatment measures and outcomes of the missions were also collected. A patient was reported as *symptom free* if the EMS team documented that “the symptom is over,” there are “no symptoms,” or the patient is in a “normal condition.” Treatment measures and vital signs measurement performed by EMS personnel were only reported from living patients that EMS personnel met at the scene. Finally, data on ED visit diagnoses, new treatment limitations, follow-up treatment and the 30-day mortality rate from EMS missions were collected from the hospital’s EHR system. Follow-up treatment was defined as an inpatient admission after an ED visit. All categories with fewer than five occurrences were classified as “other” due to practical reasons as well as patients’ confidentiality.

### Data analysis

The analyses were conducted using the available values, and missing data were not imputed. Qualitative data were classified and converted to numerical forms. The normality of data was tested using the Shapiro-Wilk test. A one-sample Chi-square test (equal distribution of missions across time periods) was used to assess the frequency distributions of EMS missions by time of day and day of the week. Differences in the distribution of EMS missions between weekdays and weekends were tested using Pearson’s Chi-square test. Statistical significance was defined as *p* < 0.05. Normally distributed patients’ ages were reported in means and standard deviations. Since most vital signs values were non-normally distributed, data on this was reported with median values, as well as with interquartile ranges (IQR) and minimum-maximum ranges (min-max). The GNU PSPP version 2.0.1 (Free Software Foundation, Boston, MA, USA) was used for all statistical analyses.

## Results

Out of 633 emergency calls, EMS personnel attended the patient on the scene in 581 cases – in the remaining 52 cases the mission was handled remotely. There were 510 patients altogether in the sample, residing in 106 nursing homes, with more than one emergency call having been made for 95 patients. The mean age of the EMS patients was 84.7 (SD 8.1), and 61.1% (*n* = 387) were women. Dementia or decline in memory function was diagnosed in 68.4% (*n* = 433) of the patients. Treatment limitations were imposed for 31.9% (*n* = 202) of the patients, and 65.9% (*n* = 417) had a DNR order. Only 10.1% had a documented advance care plans.

### Reasons for calling EMS

The majority of the missions were classified as *non-urgent* (67.7%, *n* = 433). The most common reasons for calling EMS were a fall (*n* = 169), followed by deterioration of general condition (*n* = 89), shortness of breath (*n* = 73) and chest pain (*n* = 43). The reasons for the call as recorded by EMS are shown in Table 1.

**Table 1:** Reason for the EMS call (*n* = 633)

Reason	*n*	
Fall	169	26.7%
Deterioration of general condition	89	14.1%
Shortness of breath	73	11.5%
Chest pain	43	6.8%
Altered state of consciousness	32	5.1%
Stroke	27	4.3%
Other	26	4.1%
Vomiting/diarrhea	24	3.8%
Mental health problem	21	3.3%
Sudden deterioration	18	2.8%
Cardiac arrest	17	2.7%
Pain in the limbs/body	17	2.7%
Fainting/syncope	16	2.5%
Seizure	12	1.9%
Nose bleeding	11	1.7%
Gynecological/urological bleeding	10	1.6%
Abdominal pain	9	1.4%
Backache	8	1.3%
Hematemesis	6	0.9%
Airway obstruction	5	0.8%

Prior to the emergency call, nurses had consulted their own doctor in 7.3% (*n* = 46) of the cases, and ED physician or nurse in 14.8% (*n* = 94) of the cases.

**Fig. 2:**
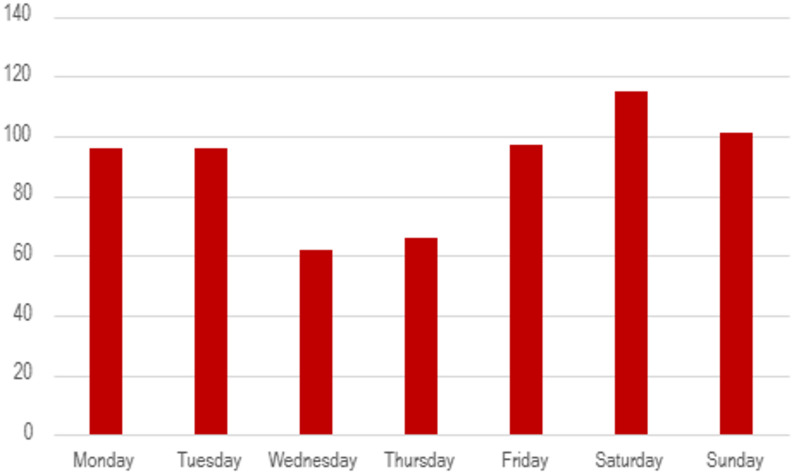
Frequency distribution of EMS missions (*n* = 633) by day of the week (χ²(6) = 24.61, *p* < 0.001)

**Fig. 3:**
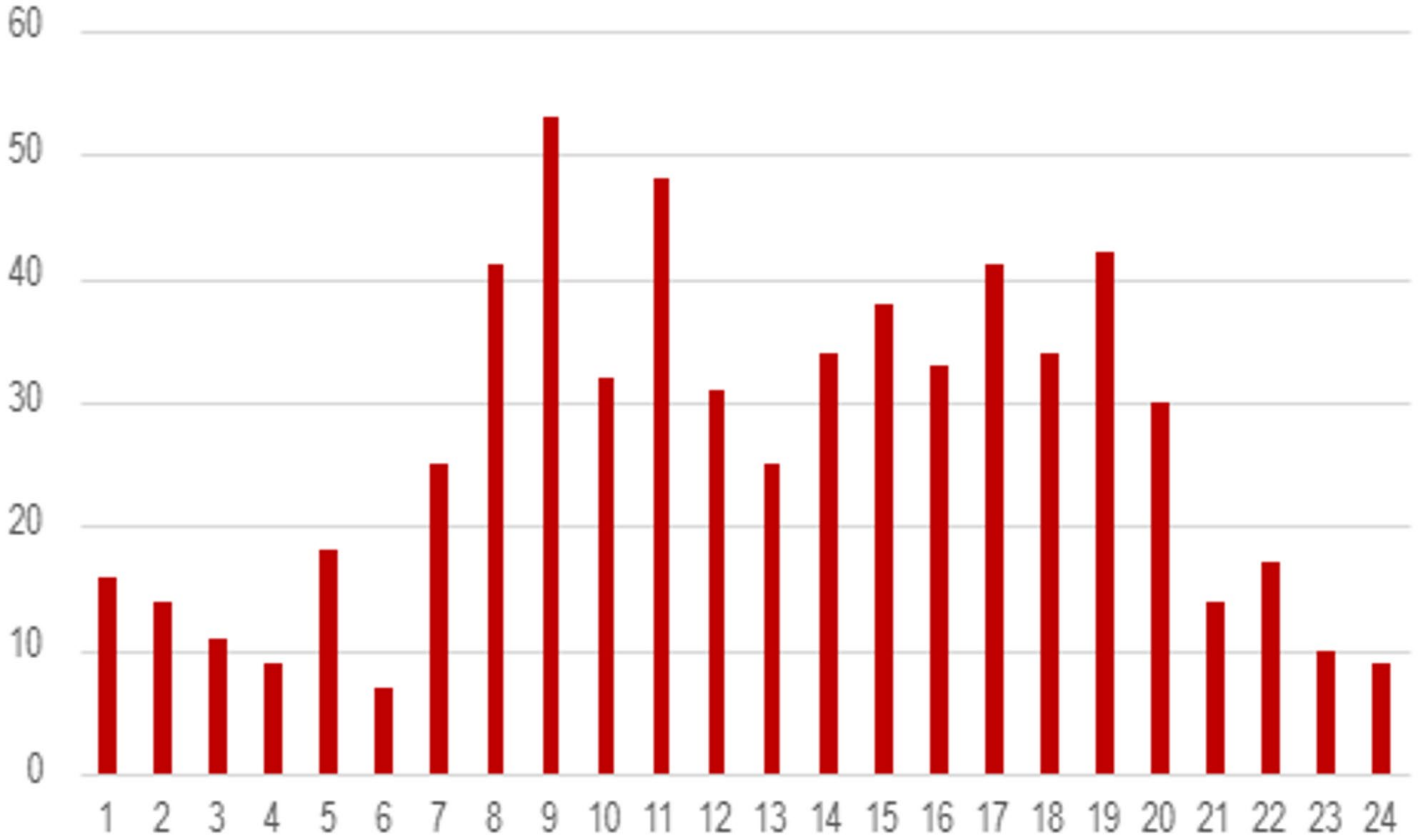
Frequency distribution of EMS missions (*n* = 633) by time of day (χ²(23) = 162.49, *p* < 0.001)

A total share of 29.4% of the EMS missions had been called in during office hours (Monday to Friday: 8 a.m. to 4 p.m.), and 34.8% during the weekend. Between day of the week and the time of day, there was a statistically significant difference in the number of EMS missions per hour (*p* < 0.001 in both cases). The greatest number of missions took place on Saturdays (*n* = 115), and least number on Wednesdays (*n* = 62). Regarding time of day, the highest number of EMS missions occurred at 9 a.m. (*n* = 53) and the lowest number at 6 a.m. (*n* = 7). There was no statistically significant difference in the distribution of missions by time between weekdays and weekends (χ²(23) = 23.02, *p* = 0.460). The distribution of missions is shown in Figs. 2 and 3.

### Treatment procedures followed prior to the emergency call

In 60.5% (*n* = 383) of the EMS missions (*n* = 633), some treatment procedures had been followed before emergency call. A share of 21% (*n* = 133) of the patients had received medication, and the most common drugs given were paracetamol (40.6%) and opioid analgesics (19.5%). The procedures performed prior to the arrival of the EMS are shown in Table 2.

**Table 2:** Treatment procedures followed prior to the arrival of EMS as a proportion of all missions (*n* = 633)

Treatment procedure	*n*	
Drug administration	133	21.0%
Vital sign measures^a^	111	17.5%
*Blood pressure*	58	9.2%
*Temperature*	52	8.2%
*Heart rate*	43	6.8%
*Oxygen saturation*	37	5.8%
C-Reactive protein (CRP) test	66	10.4%
Other test/examination	32	5.1%
Patient positioning	56	8.8%
Wound dressing	26	4.1%
Oxygen administration	7	1.1%
Cardiopulmonary resuscitation	4	0.6%

^a^ Sub-items under ‘Vital sign measures’ are not exclusive subsets; several measurements may have been recorded for an individual patient

### Treatment measures undertaken by EMS

According to the EMS teams’ assessments, the most common symptoms of living patients (*n* = 564) were pain (32.4%), shortness of breath (23.9%), and a deterioration in general condition (22.6%). In addition, 28.2% of the patients had some form of injury and 8.2% of the patients had no symptoms. Table 3 shows the first vital signs measured from the patients. The EMS set or tried to set an intravenous line for 29.2% of the patients, while 26.4% of the patients were medicated with intravenous, oral or inhaled drugs. An electrocardiogram was recorded or the heart rhythm was monitored for 25.4% of the patients, and supplemental oxygen was given to 12.9% of the patients. Additionally, some other procedures such as immobilization or wound dressing were performed on 11.2% of the patients. On 38.6% (*n* = 218) of the patients, no other treatment measures than vital sign measurements were performed.

**Table 3:** First vital signs of living patients (*n* = 564) measured by the EMS

Vital sign			Median (IQR)	Min-Max	Missing
Systolic BP** mmHg			138 (43)	42–231	*3.4%*
Diastolic BP** mmHg			75 (16)	30–159	*3.7%*
Heart rate / min			82 (26)	44–159	*3.2%*
Oxygen saturation %			96 (5)	41–100	*5.0%*
Respiratory rate / min			18 (8)	11–49	34.0%*
GCS***			15 (0)	3.0–15.0	*22.3%*
Temperature			36.80 (1)	34.3–40.1	*9.4%*
Blood sugar			7.7 (3.47)	2.1–26.6	*27.0%*

* Verbal expressions (*n* = 92) were not taken into account as missing. **Blood pressure, ***Glasgow Coma Score

### Outcomes of the calls

Of all the EMS missions, 60.3% (*n* = 382) of the patients were transferred to the ED, and 87.2% (*n* = 333) of these patients were transferred by ambulance while 12.8% were transferred by taxi. The EMS team consulted a physician in 284 missions, of which 62.3% ended up in a decision not to transfer. In 8.4% of the cases, the EMS personnel decided, with or without consulting a physician, not to transfer the patient based on the advance care plans or treatment limitations. The outcomes of the EMS missions are shown in Table 4.

**Table 4:** Outcomes of the EMS missions (*n* = 633)

	*n*	%
**Transferred**	**333**	**52.6**
Urgent	30	4.7
Non-urgent	303	47.9
**Not transferred by ambulance**	**300**	**47.4**
Guided by non-emergency transport service or taxi to emergency department	49	7.7
No further treatment needed	159	25.1
Treated on scene by EMS	72	11.4
Deceased	17	2.7
Other	3	0.5

**Fig. 4:**
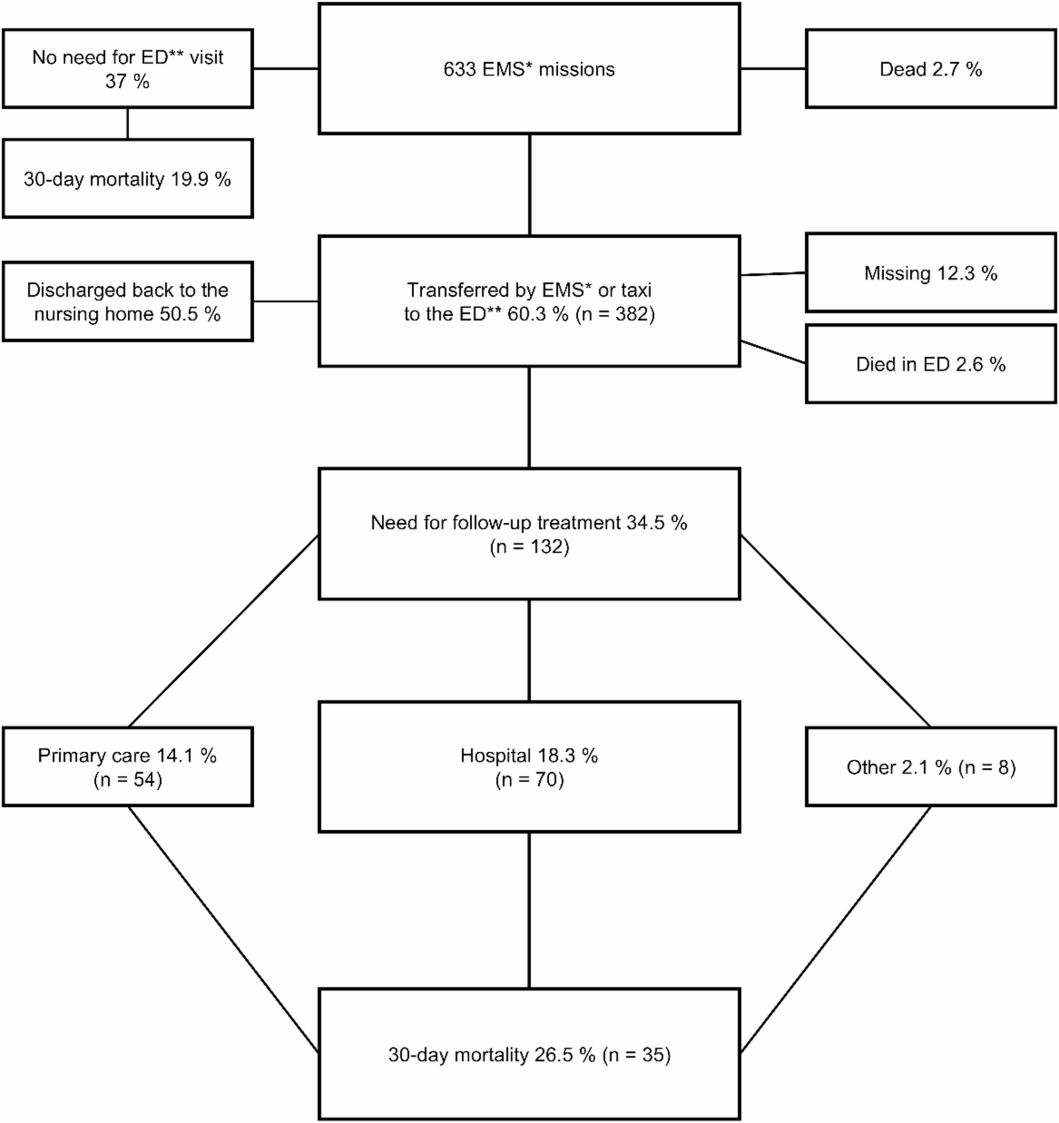
Flowchart of EMS missions (*n* = 633) outcomes *EMS = Emergency Medical Service, **ED = Emergency Department, Note: Due to data limitations, good versus poor prognosis could not be determined within the no need for ED visit -group

The most common diagnoses made during an ED visit were pneumonia (*n* = 54), hip fracture (*n* = 33), wound (*n* = 33) or cerebral hemorrhage/ stroke (*n* = 24). Of the patients who visited the ED, 27.2% (*n* = 104) did not require any treatment or medication after the examinations, 29.8% of the patients needed a course of antibiotics, 28.5% a medicine change, and 8.6% hip surgery. New treatment limitations or a DNR order were registered at the ED for 57 patients. Of all patients (*n* = 633), 20.8% (*n* = 132) required follow-up treatment. Follow-up treatments of patients after ED visits are presented in Fig. 4. The patient-specific (*n* = 510) and EMS mission-specific (*n* = 633) 30-day mortality rate was 26.5% and 25.9%, respectively. Among patients receiving follow-up treatment (*n* = 132) the 30-day mortality rate was 26.5% (*n* = 35).

## Discussion

In this study, the most common reasons for calling EMS to the nursing home were a fall, deterioration of the patient’s general condition and shortness of breath. In more than half of the missions, some treatment procedures were implemented or measurements were made before the emergency calls, and about one quarter of the patients had intravenous line treatments implemented by the EMS team or received medication from the team. About three of five patients were transferred to the ED, and one third of these patients needed follow-up treatment after their ED visits.

The findings of this study regarding reasons for calling EMS are in line with previous evidence. Several studies have shown that falls [19, 26, 27, 28, 29], a deterioration in general condition [26, 27], shortness of breath [19, 26, 27, 28] and pain are the most common situations in which older adults living in a nursing home need EMS or a visit to the ED [16]. Moreover, the need for oxygen therapy and the severity of the patient’s condition have been identified as factors associated with hospital admission [14].

In this study, in more than half of the cases covered, the nursing home staff performed more detailed measurements or treatment procedures, prior to the arrival of the EMS team. To our knowledge, no previous research on treatment procedures has been conducted. Therefore, we cannot compare the frequency of our results. However, our results indicate that nursing home nurses are generally able to assess ongoing situations in more detail. Our interpretation of nursing staff’s autonomy is limited by our inability to determine whether medications were administered according to standing orders or at the staff’s independent judgment, or whether certain procedures were performed after a physician consultation, or whether they were performed as instructed by the EMS phone service or EMS teams. In Finland, health care education is of high quality. A recent evaluation concluded that higher education (for registered nurses) provides nurses with extensive knowledge, including acute care nursing education [30]. Notwithstanding this fact, it must be emphasized that nursing home nurses often do not face acute situations in practice. During the five-month observation window for this study, the number of EMS missions per nursing home varied between 1 and 36. Thus, there may not be established routines for assessing and treating deterioration in the patients’ condition.

Previous research related to nursing home transfers has suggested that nursing home staff would often need additional resources, such as more staff, diagnostics equipment and physicians’ availability, as well as support in decision-making [31, 32]. In addition, the use of various tools such as SBAR (situation-background-action-recommendation), or National Early Warning Scoring and additional training, has slightly increased the confidence which nurses feel about their own actions [33]. Providing additional resources such as those enumerated here could strengthen the ability of nurses to handle situations more independently in the future.

The current results on the need for transfer and follow-up care in hospitals are clearly different from previous results. In this study, only about 60% of patients were transferred to the ED, compared to 78–95% in previous studies [8, 15, 17]. Since the reasons for calling EMS found in our study are consistent with previous results [19, 26, 27, 28, 29], it is likely that the lower transfer percentage is due to the different functioning of the Finnish health care system, compared to the settings in which previous studies were conducted. The transfer percentage found in the present study generally corresponds to the results of previously observed EMS transfer percentages in Finland [34–37]. In Finland, paramedics, after careful evaluation of need for care, have the opportunity to treat the patient at the scene and not to transfer if the need for an ED visit is not detected.

In addition, EMS are able to handle some of the non-urgent missions remotely [38], which also can reduce the number of possible ambulance transfers. In this study, three quarters of all decisions not to transfer had been made after a physician had been consulted. This suggests that the hospital is not considered to be the most appropriate place to care for the residents of nursing homes, and that health care professionals generally believe that their decisions should respect already established treatment limitations for patients. In other studies, advance care planning (ACP) have been found to play a role in avoiding hospitalization [39, 40]. In this study, only 10.1% of the missions’ patients had an advance care plan, although in Finland, according to the recommendation of the Ministry of Social Affairs and Health, all residents of 24-hour service housing should have an advance care plan [[41], please see pages 124–125]. The low prevalence of advance care plans may reflect systemic problems such as the low availability of physicians, the remote provision of services, the lack of a standardized ACP process, and variation in documentation practices (making advance care plan documents difficult to find and not necessarily accessible to an EMS team). However, in 55 of the cases covered in this study, EMS teams were able to decide not to transfer the patient based on an advance care plan or treatment limitations. Therefore, it can be cautiously suggested that nurses in nursing homes could have the opportunity to make similar decisions without the involvement of EMS teams, especially if advance care plans were better available.

One important finding of this study was that the proportion of patients requiring follow-up treatment was significantly lower than in previous studies [14, 21, 31, 42–44]. In Finland, nursing homes cooperate with providers of hospital services in homes, in ways that can ensure the provision of hospital-level care also at care facilities [45]. By utilizing hospital services in homes, patients thus do not actually need to stay in hospitals, since treatments like fluid therapy and IV antibiotic therapy can be arranged directly in care facilities. Together with comprehensive sets of established treatment limitations, this could explain why patients in this study required less frequent follow-up treatment.

In this study, the 30-day mortality rate was high (patient-specific 26.5% and EMS mission-specific 25.9%) although most of the missions were classified as non-urgent. This inconsistency may be due to the urgency categories assigned by the Emergency Response Centre mainly describe the urgency of the current problem, rather than the patient’s long-term prognosis. As older adults moving to a care facility in Finland typically have reduced functional status and relocate only when they require continuous 24-hour care, the 30 day follow up period is long. Consequently, high mortality may reflect the frailty and multimorbidity of this patient group rather than the severity of the missions.

Previous research has shown that avoidable ED visits cause unnecessary costs to society [46–49], as well as causing unnecessary burdens for vulnerable older adults [19, 21, 22]. While this study showed that EMS visits may have been able to prevent some unnecessary ED visits, it would be more appropriate to organize services that better address patients’ needs. In recent years in Finland, outreach acute care services have been developed, entailing that specially trained nurses can provide treatment for many acute situations outside of hospital settings [6], as well as providing remote services [38]. Such services can be used to support the operations of nursing home staff and decrease the number of EMS missions [50]. In addition, it has been cautiously suggested that outreach services are a more cost-effective solution than ED visits, and allow patients to be treated in nursing homes [6, 51].

The results of this study show that, in nursing homes, there still are situations in which residents need EMS or ED visits. In particular, the occurrence of trauma brings about situations in which ED visits cannot be avoided. Despite these exceptions, by developing different types of outreach acute care services, it is possible to support the operations of nursing home staff in non-urgent situations, and to provide older adults with more humane treatment opportunities. In line with this, we are planning to focus our future research on the nursing home staff’s views regarding resources and competencies needed to target appropriate resources in situations where the nursing home does not have sufficient ability to handle patients with deteriorating conditions.

## Strengths and limitations

The major strength of this study pertains to its coverage of a broad and heterogeneous range of nursing homes, and to its coverage of a large number of these homes. The study included all the 24-hour care facilities for the older adults in the Southwest Finland region of Finland, including units from cities, the rural and the archipelago. The nursing homes were variously organized by the wellbeing services county, private service providers or associations, and there was wide-ranging variation in the population of units. In addition, the data is comprehensive, as all EMS missions with location information and timestamps are automatically recorded into the Wellbeing Services County of Southwest Finland electronic database. The data recorded in the ePCRs was clear, and many events recorded by EMS personnel could be verified from the records of the ED.

However, this study has several limitations. Firstly, the study period exclusively covered autumn and winter. One of the most common reasons for EMS calls was shortness of breath, which is also known to be a seasonal phenomenon caused by a respiratory infection. Consequently, respiratory-related missions are likely to be overrepresented in relation to year-round activity and may introduce reporting bias. However, it should be noted that there were no missions in which the patient had fallen outdoors, thus the findings related to falls are likely unrelated to the season. Secondly, some nursing homes offer both 24-hour care facilities and sheltered housing. The type of housing was exploratively interpreted from documents by excluding missions where EMS or ED personnel mentioned a home care visit, but despite this, it is possible that some of the excluded EMS patients were residents of 24-hour care facilities. We also interpreted patients’ DNR status, ACP, treatment limitations, and dementia diagnosis from documents, and in the absence of data, it was assumed that the patient is in active treatment or that the patient does not have a memory disorder. In addition, the data was coded by one person (H.M), so the possibility of human error cannot be ruled out. Thirdly, we found large differences in the ways in which EMS personnel input data into ePCRs. Data on the patient’s respiratory rate was missing in 34% of the EMS missions although this is a very important indicator for assessing the patient’s clinical status. We also found similar results in a previous study in the same area, in which 58% of EMS mission records lacked respiratory rate data [52]. Since the majority of missions in this study were non-urgent, a possible explanation for the missing data is that a normal respiratory rate is not always recorded, or it is described verbally. Nevertheless, missing information may have limited the assessment of the patient’s deteriorated condition. In addition, some of the treatment procedures implemented prior to the EMS’s involvement had to be inferred from the documentation of the EMS phone service or the ED. Since we did not have access to nursing homes’ patient care report systems, it is possible that more patients have been treated, or that their own physicians were consulted more often by nurses, than what we could observe from the documents used in this study. Moreover, we could not reliably report whether the medications were administered prior to EMSs’ arrival based on standing orders from the nursing home or on the independent judgment of the nursing staff. Fourthly, in some cases, nurses from the ED, personnel from the EMS phone services, or EMS personnel at the mission had instructed the nursing home staff to conduct a more detailed patient assessment or to initiate treatment procedures. Thus, it remains uncertain to what extent these procedures would have been implemented without the guidance of another health care professional.

In addition, for 12.3% (*n* = 47) of the patients transferred to the ED (*n* = 382), we did not find any information about any follow-up treatment that the patients may have had. In these cases, it is possible that the patient may have been transferred to the primary health care ward without documents. Finally, the generalizability of the results is limited by the inclusion of only 24-hour care facilities. Treatment procedures performed by nurses and EMS may differ in sheltered housing without 24-hour staff or where care is provided through home care services. The generalizability is also affected by the lack of information on what kind of internal guidelines nursing homes have for managing the situations assessed in the study, as well as which treatment procedures the nursing staff can and are permitted to implement. It is possible that some nursing homes have been instructed to call the emergency number directly, which may increase the number of potentially unnecessary EMS missions. Further studies are needed on nursing homes’ internal guidelines and available treatment practices.

## Conclusions

During the study, EMSs were called to a nursing home 633 times. The majority of the missions had been defined as *non-urgent* at the Emergency Response Centre, and the most common reasons for a mission were either that a fall had occurred or that the general condition of the patient had deteriorated. The nursing staff performed some form of assessment or treatment procedure prior to EMSs’ arrival in 60.5% of the missions. Although slightly more than half of the missions resulted in an ED visit, only one-third of these patients needed hospital procedures or follow-up treatment. These findings are consistent with prior evidence suggesting that outreach acute care services and mandatory ACP documentation may be associated with reductions in EMS utilization. Prospective studies are needed to determine whether such interventions would reduce unnecessary missions and transfers in this context.

## Data Availability

The datasets generated and analyzed in the current study are not publicly available, due to legal and ethical restrictions related to the use of sensitive personal data.

## Abbreviations

ACP: Advance care planning
DNR: Do not resuscitate
ED: Emergency department
EHR: Electronic health record
EMS: Emergency medical services
ePCR: Electronic patient care reports
GCS: Glasgow Coma Scale
SBAR: Situation-background-action-recommendation
